# Impact of peripheral mitochondrial DNA level on immune response after COVID-19 vaccination

**DOI:** 10.1016/j.isci.2023.107094

**Published:** 2023-06-10

**Authors:** Hiroaki Ikezaki, Hideyuki Nomura, Nobuyuki Shimono

**Affiliations:** 1 Department of General Internal Medicine, Kyushu University Hospital, Fukuoka 8128582, Japan; 2 Department of Comprehensive General Internal Medicine, Kyushu University Faculty of Medical Sciences, Fukuoka 8128582, Japan; 3 Department of Internal Medicine, Haradoi Hospital, Fukuoka 8138588, Japan

**Keywords:** Immunology, Virology

## Abstract

The efficacy of vaccination against severe acute respiratory syndrome coronavirus 2 (SARS-CoV-2) in the elderly is partially hindered by immunosenescence, resulting from decreased mtDNA levels. This study evaluated the correlation between mtDNA levels in peripheral leukocytes and immune response to the SARS-CoV-2 vaccine. Two hundred ten participants (median age 79.5 years), including 83 frail residents/inpatients and 70 robust outpatients, were analyzed. Anti-spike IgG antibody (IgG(S)) titers were serially measured from before the first vaccination to after the third vaccination. The mtDNA levels and cell-mediated immunity were measured in 45 elderly and 22 elderly individuals two months after the third vaccination. The robust group had consistently higher IgG(S) titers than the frail group. The mtDNA levels positively correlated with IgG(S) titers, as well as with cell-mediated immunity. These findings suggest that mtDNA levels positively impact vaccine-induced immunity. Further studies into maintaining mtDNA levels may provide insights into immunosenescence in the elderly.

## Highlights

• Peripheral mitochondrial DNA levels (mtDNA-CN) were assessed in the older people
• The mtDNA-CN positively correlated with cell-mediated immune response
• The mtDNA-CN also positively correlated with humoral immunity
• The mtDNA-CN may be an indicator of vaccine efficacy or immunosenescence

## Graphical abstract

**Figure:**
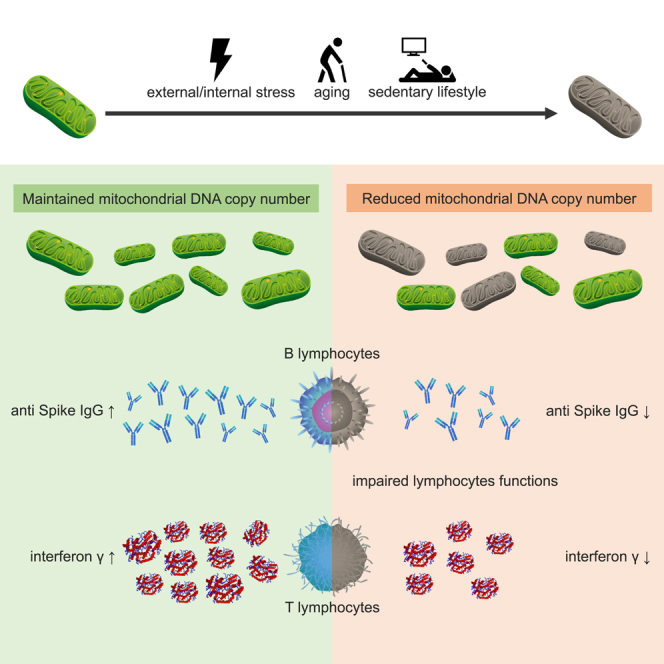

## Introduction

The ongoing COVID-19 pandemic has wrought widespread devastation, particularly among the elderly.^1^^,^^2^ Despite the deployment of vaccines against severe acute respiratory syndrome coronavirus 2 (SARS-CoV-2), the elderly population remains at an elevated risk of infection, hospitalization, and mortality. One contributing factor is the emergence of highly transmissible, immune-evasive variants of the virus, such as the omicron variant, which features numerous mutations in the spike protein’s receptor-binding domain.^3^^,^^4^^,^^5^ Additionally, the efficacy of vaccine-induced immunity has been demonstrated to wane over time.^6^^,^^7^ To address this waning immunity, booster doses have been recommended.^8^^,^^9^

Another issue is immunosenescence, characterized by a gradual degradation of the immune system in the elderly population and an increased susceptibility to infectious diseases. It has been reported that vaccine-induced anti-spike immunoglobulin G (IgG) titers in elderly individuals are lower than those in younger individuals.^10^^,^^11^ The prominent characteristic of immunosenescence is a reduction in the number and function of T and B lymphocytes. In addition, inflammaging, a chronic, low-grade inflammation, induces immunosenescence.^12^ Recent research has suggested that a decline or dysfunction in intracellular mitochondria may contribute to these age-associated degradations of T and B lymphocyte functions and inflammaging.^13^ Thus, mitochondria may play a crucial role in the immune response to both infection and vaccination, particularly in the elderly population. However, it has yet to be established that assessing mitochondrial levels and functions with a less invasive method. Recently, it has been reported that the number of intracellular mitochondria, measured by mitochondrial DNA (mtDNA) copy number (mtDNA-CN) in peripheral leukocytes, decreases with aging and illness.^14^^,^^15^^,^^16^ This study aims to investigate the relationship between mitochondrial DNA copy number in peripheral leukocytes and the immune response to SARS-CoV-2 vaccination.

## Results

Table S1 shows the baseline characteristics of the 198 analyzed participants. The median age was 38 years for the Young group, 78 years for the Robust group, and 90 years for the Frail group. Because most of the Young group were nurses, the proportion of women was higher in the Young group. There was no significant difference in smoking habits or allergies. Alcohol drinking habits were more common in the Young and Robust groups than in the Frail group. The Robust and Frail groups had more comorbidities than the Young group. Of the serum measurements, the Frail group had significantly lower total bilirubin and ALT levels than the other groups. The Robust group had substantially higher AST, ALT, and γ-GTP levels than the other groups. The Young group had significantly lower serum creatinine levels and higher eGFR than the other groups.

The dynamics of the SARS-CoV-2 anti-spike IgG titers are shown in Figure S1. Participants who were found to have COVID-19 infection (e.g., positive for PCR or anti-nucleocapsid IgG antibody) were excluded from the analysis. As we have previously reported, vaccine-induced anti-spike IgG levels in the Young group were significantly and twice as high as those in the Robust group and five to ten times higher than those in the Frail group across time points until the third vaccination. Anti-spike IgG levels in the Robust group were also significantly and twice to four times higher than those in the Frail group until the third vaccination. However, anti-spike IgG levels six months after the second vaccination considerably decreased to 1000 AU/ml in the Young group, 500 AU/ml in the Robust group, and 200 AU/ml in the Frail group. A significant increase in anti-spike IgG levels was observed after the third vaccination, 20-fold in the Young group, 40-fold in the Robust group, and 50-fold in the Frail group. After the third vaccination, the Robust group showed the highest anti-spike IgG levels, followed by the Young group and then the Frail group (21642, 17508, and 15529 AU/ml, respectively, P for trend 0.06). This trend remained until six months after the third vaccination. Anti-spike IgG levels six months after the third vaccination were 5944 AU/ml in the Robust group, 3662 AU/ml in the Young group, and 3321 AU/ml in the Frail group (P for trend <0.01).

**Figure 1:**
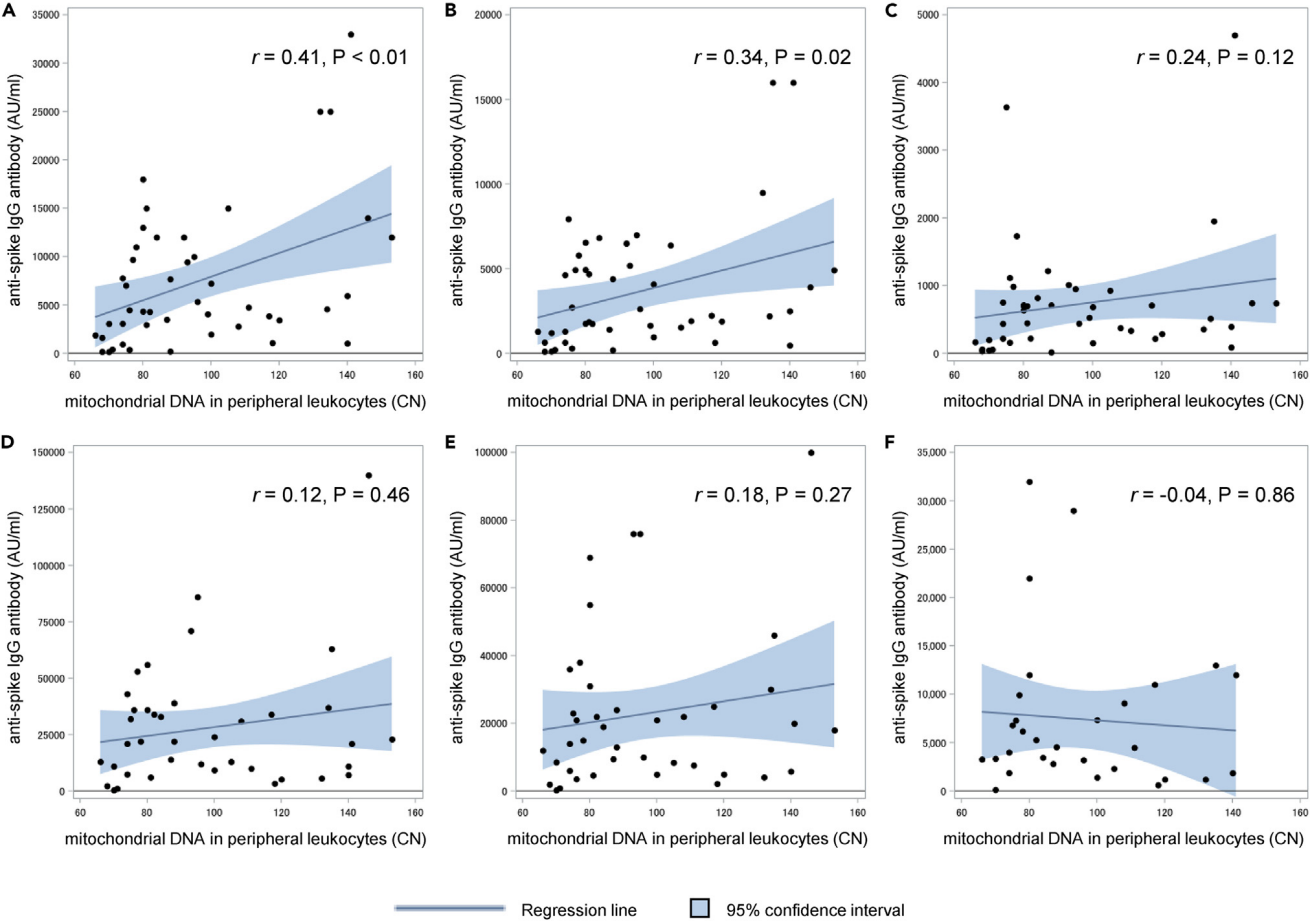
The correlations between peripheral mitochondrial DNA copy number and anti-spike IgG titers across time points Black circles represented each participant. The solid blue line shows the regression line, and the light blue area shows the 95% confidence interval. Figures show the correlations at one month (1A), two months (1B), and six months (1C) after the second vaccination, and at one month (1D), two months (1E), and six months (1F) after the third vaccination. Peripheral mitochondrial DNA copy number levels were measured two months after the third vaccination.

We assessed the correlation between mtDNA-CN and anti-spike IgG levels among 45 participants (35 from the Robust group and ten from the Frail group) who gave additional informed consent for measurement of mtDNA-CN. Table 1 shows the characteristics of the 45 analyzed participants. All Frail participants received mRNA-1273 as the third vaccination. Among the Robust participants, 14 (40.0%) received mRNA-1273, and the rest received BNT162b2 as the third vaccination. Median mtDNA-CN was significantly higher in the Robust group than in the Frail group. The Robust group was eight years younger than the Frail group, and one-third were male. The participants in the Robust group were more likely to have a drinking habit and had higher levels of total bilirubin and AST. In this population, mtDNA-CN showed a significantly negative correlation with age (r = −0.30, p = 0.04). Scatterplots of the correlation between mtDNA-CN at two months after the third vaccination and anti-spike IgG levels across the time points are shown in Figure 1. The mtDNA-CN showed significant but weak positive correlations with IgG levels one and two months after the second vaccination (Figures 1A and 1B, *r* = 0.41 and 0.34, p < 0.01 and p = 0.02, respectively). However, no significant correlation was observed six months after the second vaccination or after the third vaccination (Figures 1C–1F). This analyzed population had no significant correlation between age and anti-spike IgG levels.

**Table 1:** Characteristics of participants in the mitochondrial DNA copy number and cell-mediated immunity analysis

	Robust^a^ (n = 35)	Frail^b^ (n = 10)
Demographic		
Age – years	81 [77, 87]	89.5 [79, 96]
Sex – no. (%)		
Female	23 (65.7)	9 (90.0)
Male	12 (34.3)	1 (10.0)
Smoking habit – no. (current/past/never)	1/6/28	0/0/10
Alcohol drinking habit – no. (daily/often/never)	5/9/21	0/0/10
Allergy – no. (%)	4 (11.4)	3 (30.0)
Number of comorbidities – no.	3 [2, 4]	3 [2, 4]

Type of the third vaccination – no. (%)		

Pfizer	21 (60.0)	0 (0.0)
Moderna	14 (40.0)	10 (100)

Laboratory measurement		

Mitochondrial DNA – copy number	95 [80, 132]	76.5 [70, 84]
Ag1 (CD4) – Nil (IU/mL)	0.17 [0.085, 0.66]	0.075 [0.00, 0.10]
Ag2 (CD4/CD8) – Nil (IU/mL)	0.30 [0.075, 0.765]	0.08 [0.04, 0.23]
Total bilirubin – mg/dl	0.6 [0.4, 0.8]	0.3 [0.2, 0.3]
Aspartate aminotransferase – IU/ml	21 [19, 26]	16 [13, 23]
Alanine aminotransferase – IU/ml	15 [12, 20]	11.5 [8, 21]
γ-glutamyl transpeptidase – IU/ml	21 [16, 38]	18.5 [13, 36]
Serum creatinine – mg/dl	0.81 [0.65, 1.06]	0.75 [0.60, 0.97]
eGFR – ml/min/m^2^	59.4 [47.4, 69.8]	62.6 [42.5, 77.2]

ADL, activities of daily living; eGFR, estimated glomerular filtration rate; IU, international unit.

a Robust group consists of outpatients and medical staff who are older than 60 years and have independent ADL.

b Frail group consists of nursing home residents and inpatients of long-term care units.

**Figure 2:**
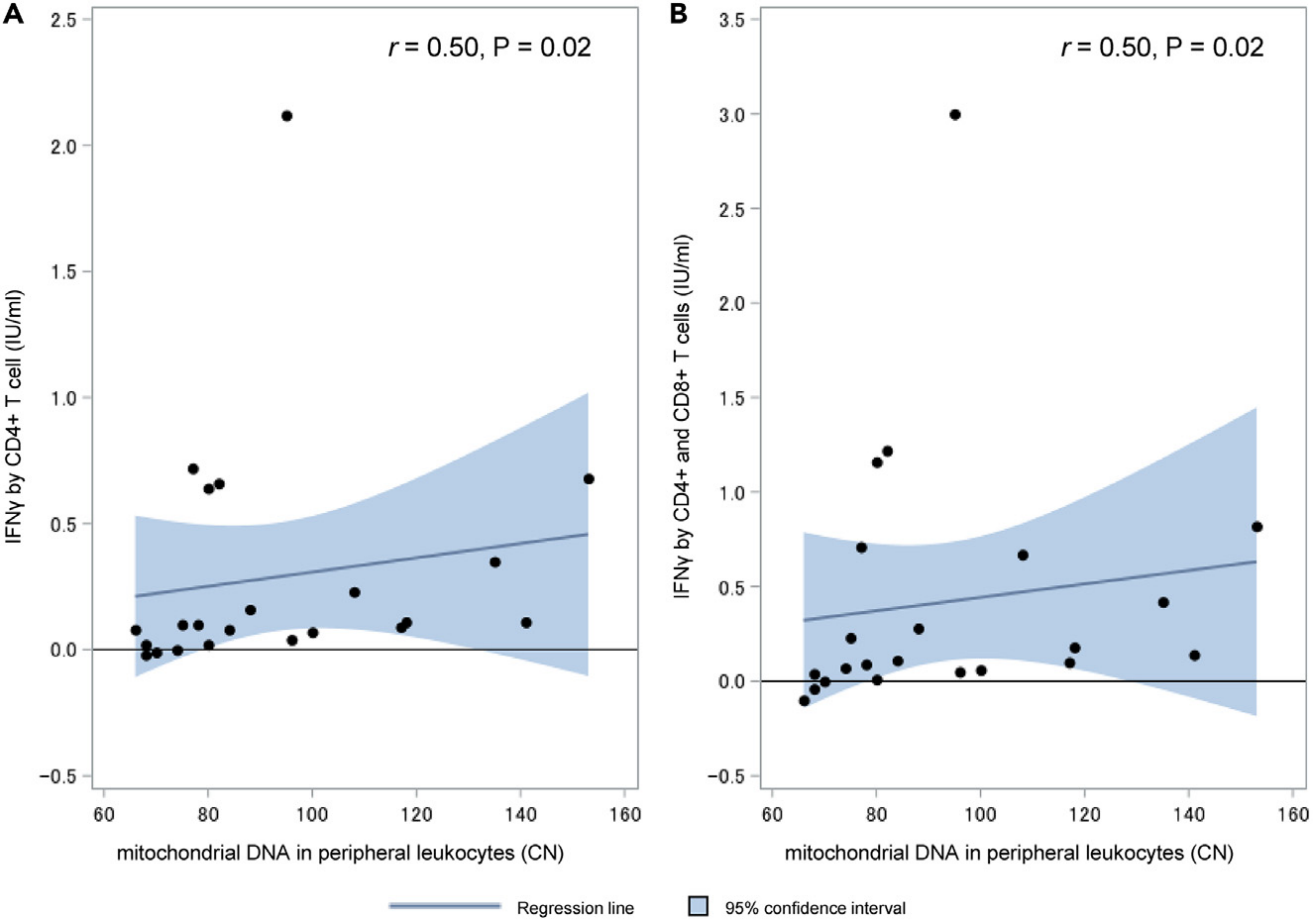
The correlations between peripheral mitochondrial DNA copy number and cell-mediated immune responses at two months after the third vaccination Black circles represented each participant. The solid blue line shows the regression line, and the light blue area shows the 95% confidence interval. Figure 2A shows the correlation between peripheral mitochondrial DNA copy number and interferon γ levels induced by CD4^+^ T cell. Figure 2B shows the correlation between peripheral mitochondrial DNA copy number and interferon γ levels induced by CD4^+^ and CD8^+^ T cells.

In addition, we assessed the correlation between mtDNA-CN and cell-mediated immunity against SARS-CoV-2 among 22 participants (12 from the Robust group and ten from the Frail group) who gave additional informed consent for measurements of mtDNA-CN and cell-mediated immunity. The characteristics of the 22 analyzed participants are shown in Table 2. In this analysis, all participants from the Robust group were female. All Frail participants received mRNA-1273 as the third vaccination. Among the Robust participants, 7 (58.3%) received mRNA-1273; the rest received BNT162b2 as the third vaccination. The Robust group had a significantly higher median mtDNA-CN and CD4^+^ T cell response to SARS-CoV-2 than the Frail group. In this population, there was no correlation between mtDNA-CN and age. The correlations between mtDNA-CN and cell-mediated immunity two months after the third vaccination are shown in Figure 2. Significant positive correlations were found between mtDNA-CN and both CD4^+^ and CD4^+^/CD8^+^ T cell responses (both *r* = 0.50 and p = 0.02, respectively). There was no significant correlation between age and cell-mediated immunity in this analyzed population. The correlations between mtDNA-CN and both CD4^+^ and CD4^+^/CD8^+^ T cell responses remained significantly positive even after adjusting for age and sex (*r* = 0.49 and 0.50, both p = 0.03, respectively). In addition, the correlations between cell-mediated immunity and anti-spike IgG levels one and two months after the third vaccination are shown in Figure S2. CD4^+^ and CD4^+^/CD8^+^ T cell responses showed significantly positive correlations with anti-spike IgG levels. Those correlations were not significant at other time points (data are not shown).

**Table 2:** Characteristics of participants in the cell-mediated immunity against SARS-CoV-2 analysis

	Robust^a^ (n = 12)	Frail^b^ (n = 10)
Demographic		
Age – years	81 [78.5, 85.5]	89.5 [79, 96]
Sex – no. (%)		
Female	12 (100)	9 (90.0)
Male	0 (0.0)	1 (10.0)
Smoking habit – no. (current/past/never)	0/1/11	0/0/10
Alcohol drinking habit – no. (daily/often/never)	0/4/8	0/0/10
Allergy – no. (%)	2 (16.7)	3 (30.0)
Number of comorbidities – no.	3 [1, 4]	3 [2, 4]

Type of the third vaccination – no. (%)		

Pfizer	5 (41.7)	0 (0.0)
Moderna	7 (58.3)	10 (100)

Laboratory measurement		

Mitochondrial DNA – copy number	112.5 [80, 138]	76.5 [70, 84]
Ag1 (CD4) – Nil (IU/mL)	0.17 [0.085, 0.66]	0.075 [0.00, 0.10]
Ag2 (CD4/CD8) – Nil (IU/mL)	0.30 [0.075, 0.765]	0.08 [0.04, 0.23]
Total bilirubin – mg/dl	0.65 [0.4, 0.8]	0.3 [0.2, 0.3]
Aspartate aminotransferase – IU/ml	22.5 [20, 30]	16 [13, 23]
Alanine aminotransferase – IU/ml	15 [13, 25.5]	11.5 [8, 21]
γ-glutamyl transpeptidase – IU/ml	19 [15, 33.5]	18.5 [13, 36]
Serum creatinine – mg/dl	0.72 [0.63, 0.885]	0.745 [0.60, 0.97]
eGFR – ml/min/m^2^	57.8 [47.1, 68.9]	62.6 [42.5, 77.2]

ADL, activities of daily living; eGFR, estimated glomerular filtration rate; IU, international unit.

a Robust group consists of outpatients and medical staff who are older than 60 years and have independent ADL.

b Frail group consists of nursing home residents and inpatients of long-term care units.

## Discussion

Our study reveals a statistically significant positive correlation between mtDNA-CN levels in peripheral leukocytes and vaccine-mediated cellular immunity in older individuals. This correlation remained robust even after controlling for confounding factors such as age and sex. No significant relationship was observed between age and cellular immunity. Based on these results, we posit that mtDNA-CN serves as a reliable predictor of immunosenescence, a state of diminishing immunity that poses a significant risk to elderly individuals, particularly in terms of their vulnerability to severe infections and reduced vaccine efficacy. Our results suggest that maintaining mtDNA-CN levels in the elderly may thus prevent the development of immunosenescence and improve COVID-19 outcomes.

Mitochondria are multifunctional intracellular organelles and are involved in cellular homeostasis, metabolism, and innate immune signaling. They play a central role in innate immunity against many diseases, especially infectious diseases.^17^^,^^18^^,^^19^ Activating the immune response through mitochondrial antiviral signaling (MAVS), a protein located on the outer mitochondrial membrane, is essential for the innate immunity against viral infections.^20^ The transduction of nucleic acid-derived information from infectious viruses to MAVS induces the production of type 1 interferon and various proinflammatory cytokines, which play crucial roles in the antiviral response.^20^ In addition, the innate immune responses are elicited by mitochondrial damage-associated molecular patterns, such as N-formyl peptides, cytochrome *c*, cardiolipin, and fragments of mtDNA, which are released from injured or dying cells. The mtDNA fragments released into the cytoplasm or circulation induce the transcription of various interferon stimulatory genes, which have antiviral dispositions.^21^ Inflammasome activation is another pathway through which mitochondria are involved in the immune response. Inflammasomes are multiprotein oligomers located in the cytoplasm that act as innate immune system receptors and regulate caspase-1 activation and interleukin secretion.^22^ Inflammasome activation inhibits ATP production in the mitochondria, which leads to the acceleration of reactive oxygen species (ROS) production in the mitochondria. Increasing ROS production in mitochondria causes mitochondrial dysfunction, which damages mtDNA. Subsequently, damaged mtDNA is released into the cytoplasm, and inflammasomes are further activated.^23^

Chronic inflammation alters the quality and quantity of mtDNA, and continuous mitochondrial dysfunction decreases mtDNA-CN. It is also known that aging strongly affects mitochondrial dysfunction by accumulating mtDNA mutations.^24^ As a consequence, mtDNA levels decline, and immunosenescence is elicited. It has been reported that mtDNA-CN is a surrogate biomarker of mitochondrial dysfunction^25^ and is associated with various diseases. Decreased mtDNA-CN predicted the incidence of cardiovascular disease and mediated the effect of type 2 diabetes mellitus on cardiovascular disease risk among middle-aged women.^16^ It has also been reported that lower mtDNA-CN is associated with a higher risk of type 2 diabetes mellitus, independent of age, BMI, smoking status, and physical activity.^26^ Regarding COVID-19, decreased peripheral mtDNA levels were reported to be associated with severity and mortality.^27^ Our results are consistent with previous studies; decreased mtDNA levels are associated with immunosenescence.

T lymphocytes are one of the essential white blood cells of the immune system and play a central role in the adaptive immune response, including defense against viral infection and vaccine effectiveness. T lymphocytes secrete a range of cytokines and mediators, including IFN-γ, one of the most important cytokines. Mitochondria play essential and diverse roles in T lymphocyte development, metabolism, and activation. Mitochondrial ATP production is necessary for migration when T lymphocytes detect chemotactic factors.^28^ Local mitochondrial ATP and calcium buffering are also needed at the immune synapse.^29^ Mitochondrial ROS signals are fundamental for T lymphocyte activation and proliferation. Mitochondrial metabolism regulates the differentiation of T lymphocytes into cytotoxic, effector, or regulatory subsets. The fusion of mitochondria promotes a small population of the long-living phenotype of memory cells to survive.^30^ Th1 cells are one of the effector subsets that differentiate from CD4^+^ T lymphocytes and secrete IFN-γ.^30^ Our results showed that decreased mtDNA-CN was associated with lower responses of Th1 cells, which is consistent with the concept that mitochondria are essential for T lymphocyte adaptive responses.

B lymphocytes are another type of essential white blood cell and are responsible for protective antibody production. Mitochondria also play an important role in B lymphocyte development, activation, and differentiation, as well as in T lymphocytes. Naive B lymphocytes activated by cognate antigens migrate to secondary lymphoid tissues called germinal centers.^31^ Activated B lymphocytes undergo class switch recombination to different immunoglobulin classes, editing their immunoglobulin genes to improve their affinity to the antigen in the germinal centers.^31^ Increased mitochondrial content is reported to enhance B lymphocyte differentiation through class switch recombination.^32^ This process generates functionally distinct B lymphocyte subsets, including plasma cells that produce IgG.^32^ In our study, decreased mtDNA-CN was associated with lower anti-spike IgG antibody levels after the first and second vaccinations. Our results suggest that mtDNA-CN affects antigen-specific IgG production in the early stage of antigen invasion.

In conclusion, our study presented lower levels of mtDNA-CN associated with lower cell-mediated immunity induced by vaccination. Thus, maintaining mitochondrial integrity is essential for sufficient immune system responses against vaccination. Further studies are necessary to elucidate effective therapeutic approaches, including lifestyle habits, to maintain mtDNA-CN.

### Limitations of the study

This study has some notable limitations. First, our cohorts were small in size, especially the cohort evaluating cell-mediated immunity. In addition, our cohorts consisted of Japanese participants, and mtDNA-CN was assessed only in elderly individuals. However, the fact that our participants were a very elderly population with a mean age of over 80 years is a strength of this study. Further studies of more diverse populations could assess the effect of mtDNA-CN on immune responses based on differences in sex, age, or ethnicity. Second, mtDNA-CN was measured two months after the third vaccination. Therefore, the study design is cross-sectional. Prospective studies are necessary to assess the influence of mtDNA-CN on adaptive immunity. Third, few participants in our cohorts were infected by SARS-CoV-2. Moreover, none of the mtDNA-CN cohort was infected. Therefore, we could not evaluate whether mtDNA-CN levels affect the prevention of SARS-CoV-2 infection or the severity of COVID-19. Further studies with a large population and more extended observation periods are warranted.

## STAR★Methods

### Key resources table

**Table:** 

REAGENT or RESOURCE	SOURCE	IDENTIFIER
**Biological samples**		

Whole blood samples of 45 participants	This paper	N/A
Plasma of 198 participants	This paper	N/A

**Critical commercial assays**		

SARS-CoV-2 IgG II Quant assay	Abbott Diagnostics	Cat# 6S60 472062R03
SARS-CoV-2 IgG	Abbott Diagnostics	Cat# 6R86 471922R03
SARS-CoV-2 IgM	Abbott Diagnostics	Cat# 6R87 472084R03
QuantiFERON SARS-CoV-2 Starter Pack	Qiagen	Cat# 626715

**Software and algorithms**		

SAS version 9.4	SAS Institute	https://www.sas.com

### Resource availability

#### Lead contact

Further information and requests for resources and reagents should be directed to and will be fulfilled by the lead contact, Hiroaki Ikezaki (ikezaki.hiroaki.149@m.kyushu-u.ac.jp).

#### Materials availability

This study did not generate new unique reagents.

### Experimental model and subject details

#### Study participants and collection of clinical samples

Study participants were recruited from among health care workers, outpatients, and inpatients of long-term care units in Haradoi Hospital, a care-mix hospital in Fukuoka. In addition, residents of nursing homes affiliated with Haradoi Hospital were recruited. The recruitment for this study was conducted from March 14, 2021, to July 30, 2021, and the blood samples were collected from March 21, 2021, to October 14, 2022.^6^^,^^10^ In total, 210 individuals (median age 79.5 years, 147 women) participated in this long-term prospective study. We categorized participants into three groups: 83 nursing home residents or inpatients of long-term care units older than 60 years as the Frail group, 70 outpatients or medical staff more aged than 60 years as the Robust group, and 57 medical staff younger than 60 years as the Young group. All participants were offered first and second doses of BNT162b2 (Comirnaty®: Pfizer/BioNTech) between March and August 2021. A booster (third) dose of vaccine was offered between December 2021 and April 2022. The offered booster vaccine was BNT162b2 or mRNA-1273 (Spikevax®: Moderna). Participants provided information on their height, weight, smoking habits (current, past, or never), drinking habits (daily, often, or never), allergies, medical history, medications, whether they had adverse reactions to vaccination (such as fever), and whether they had needed antipyretics. To evaluate the dynamics of anti-spike IgG titers after vaccination, blood samples were collected at the following timepoints: before the first vaccination; three weeks after the first vaccination (just before the second vaccination); one, two, and six months after the second vaccination; and one, two, and six months after the third vaccination. In addition, mtDNA-CN and cell-mediated immunity against SARS-CoV-2 were assessed in participants who gave another informed consent using blood samples at two months after the third vaccination (Figure S3).

This study was carried out in accordance with the principles of the Declaration of Helsinki, as revised in 2008. This study was approved by the Haradoi Hospital institutional ethics review committee prior to data collection (Approval No. 2020-08), and all participants provided written informed consent prior to enrollment.

### Method details

#### Assessment of SARS-CoV-2 antibody responses

Levels of anti-spike IgG were quantified using a SARS-CoV-2 IgG II Quant assay (Abbott Diagnostics, Chicago, IL, USA). The results of anti-spike IgG quantification are expressed as arbitrary units per milliliter (AU/ml) (positive threshold: 50 AU/ml). We also performed qualitative tests for IgG/immunoglobulin M (IgM) antibodies against the SARS-CoV-2 nucleocapsid protein (positive thresholds: 1.40 index [S/C] for anti-nucleocapsid IgG and 1.00 index [S/C] for anti-nucleocapsid IgM) for all participants to exclude the effects of SARS-CoV-2 infection. Participants also had blood tests for total bilirubin, aspartate aminotransferase (AST), alanine aminotransferase (ALT), γ-glutamyl transpeptidase (γ-GTP), and serum creatinine levels using standard enzymatic methods. The estimated glomerular filtration rate (eGFR) was calculated using the following equation: 194 × serum creatinine-1.094 × age-0.287 (× 0.739 [if female]).

#### Measurement of mitochondrial DNA copy number

Peripheral whole blood samples obtained in a standard tube with an anticoagulant (EDTA) were used to determine the mtDNA-CN. Total genomic DNA was extracted from whole blood using the QIAamp DNA kit (Qiagen, Germantown, MD). The concentration of extracted DNA was quantified and adjusted to 20ng/μl for measuring both mtDNA and nuclear DNA (nDNA). The above process was performed within 24 hours of blood collection. The extracted DNA samples were stored in a refrigerator at around 4°C until measurement within a few days. We utilized target sequences from the NADH dehydrogenase 1 (ND1) and cytochrome B (CYB) regions of the mitochondrial genome as two sets of primers and probes for mtDNA quantification. For nDNA quantification, a primer and probe targeting the Serine Protease Inhibitor-A1 (SERPINA1) were selected. For digital PCR and TaqMan PCR, the following primers were used:

ND1: Forward: 5′-CCCTAAAACCCGCCACATCT-3′

 Reverse: 5′-GAGCGATGGTGAGAGCTAAGGT-3′

CYB: Forward: 5′-CTCACTCCTTGGCGCCTGCC-3′

 Reverse: 5′-GGCGGTTGAGGCGTCTGGTG-3′

SERPINA1: Forward: 5′-TTTTGGTTTAGTTTAGGATTTTGAGG-3′

 Reverse: 5′-ACCTACCAATTATTAATACCAAATCTATAC-3′

A digital PCR was performed on a QuantStudio 3D Digital PCR platform (Thermo Fisher Scientific Inc., Waltham, MA, USA) with the following conditions: 96°C for 10 min, 40 cycles of 98°C for 30 s, and 60°C for 2 min. A TaqMan PCR was performed on a LightCycler® 480 Real-Time PCR System (Roche Diagnostics K. K., Tokyo, Japan) with the following conditions: 95°C for 5 min, 40 cycles of 95°C for 15 s, and 60°C for 1 min. Three samples arbitrarily chosen from the extracted DNA samples were used to quantify the expression of ND1, CYB, and SERPINA1 by digital PCR to prepare standard DNA and used as external controls. The samples used to determine the external control were measured simultaneously at each measurement time to eliminate inter-assay variations. A 10-fold dilution series of the external control was used to generate a calibration curve for TaqMan PCR. ND1 and CYB were each divided by the value of SERPINA1, and the higher value was used as the mtDNA-CN.

#### Assessment of cell-mediated immunity

Cell-mediated immunity against SARS-CoV-2 was assessed using the QuantiFERON SARS-CoV-2 assay kit (QuantiFERON® SARS-CoV-2 Starter Set Blood Collection Tubes; Qiagen, Germantown, MD). This assay evaluates the CD4^+^ and CD8^+^ T lymphocyte responses and consists of four tubes, Nil, Mitogen, SARS-CoV-2 Antigen 1 (Ag1), and SARS-CoV-2 Antigen 2 (Ag2). The SARS-CoV-2 Ag1 tube contains epitopes derived from the S1 subunit (RBD; Receptor Binding Domain) of the Spike protein, which are recognized mainly by CD4^+^ T lymphocytes. The SARS-CoV-2 Antigen 2 (Ag2) tube contains epitopes derived from the Spike protein, which CD4^+^ and CD8^+^ T lymphocytes recognize. Nil and Mitogen are intended to be used as negative and positive controls, respectively. Samples were processed according to the manufacturer's guidelines.^33^^,^^34^^,^^35^ Briefly, whole blood samples were collected in lithium heparin tubes and dispensed into the four tubes coated with Ag1, Ag2, and phytohemagglutinin as the positive control and no peptides as the negative control, respectively. After incubation at 37°C for 24 hours, the supernatants collected from the cultured whole blood samples were subjected to an enzyme-linked immunosorbent assay (ELISA)-based platform to measure interferon-gamma (IFN-γ) concentration.

### Quantification and statistical analysis

#### Statistical analysis

Data are expressed as median values with 25th and 75th percentile values for continuous variables. The geometric mean titers (GMTs) of anti-spike IgG were calculated. Categorical variables are reported as frequencies and percentages. The Mann–Whitney U test was used to compare two groups, and the Kruskal–Wallis test was used to compare three groups. The Tukey–Kramer method was used for each two-group comparison among the three groups. Anti-spike IgG levels, with adjustment for age and sex, were determined by the least means square method. All analyses were performed using SAS version 9.4 (SAS Institute Inc., Cary, NC). A P value less than 0.05 was considered to indicate statistical significance.

## Data Availability

• The data supporting the findings of this study are available within the paper and the raw data available from the lead contact upon request and with the appropriate and approved data sharing agreement. • This paper does not report original code. • Any additional information required to reanalyze the data reported in this paper is available from the lead contact upon request.
