# Supplementary material for: Impact of peripheral mitochondrial DNA level on immune response after COVID-19 vaccination
Source: iScience. 2023 Jun 10;26(7):107094. doi: 10.1016/j.isci.2023.107094 (PMC10256584; doi:10.1016/j.isci.2023.107094)
Supplement: Document S1. Figures S1–S3 and Table S1 [file mmc1.pdf]

iScience, Volume *26*

Supplemental information

Impact of peripheral mitochondrial DNA level on immune response after COVID-19 vaccination

Hiroaki Ikezaki, Hideyuki Nomura, and Nobuyuki Shimono

Figure S1. The dynamics of the anti-spike IgG titers across timepoints. (Related to Figure 1)

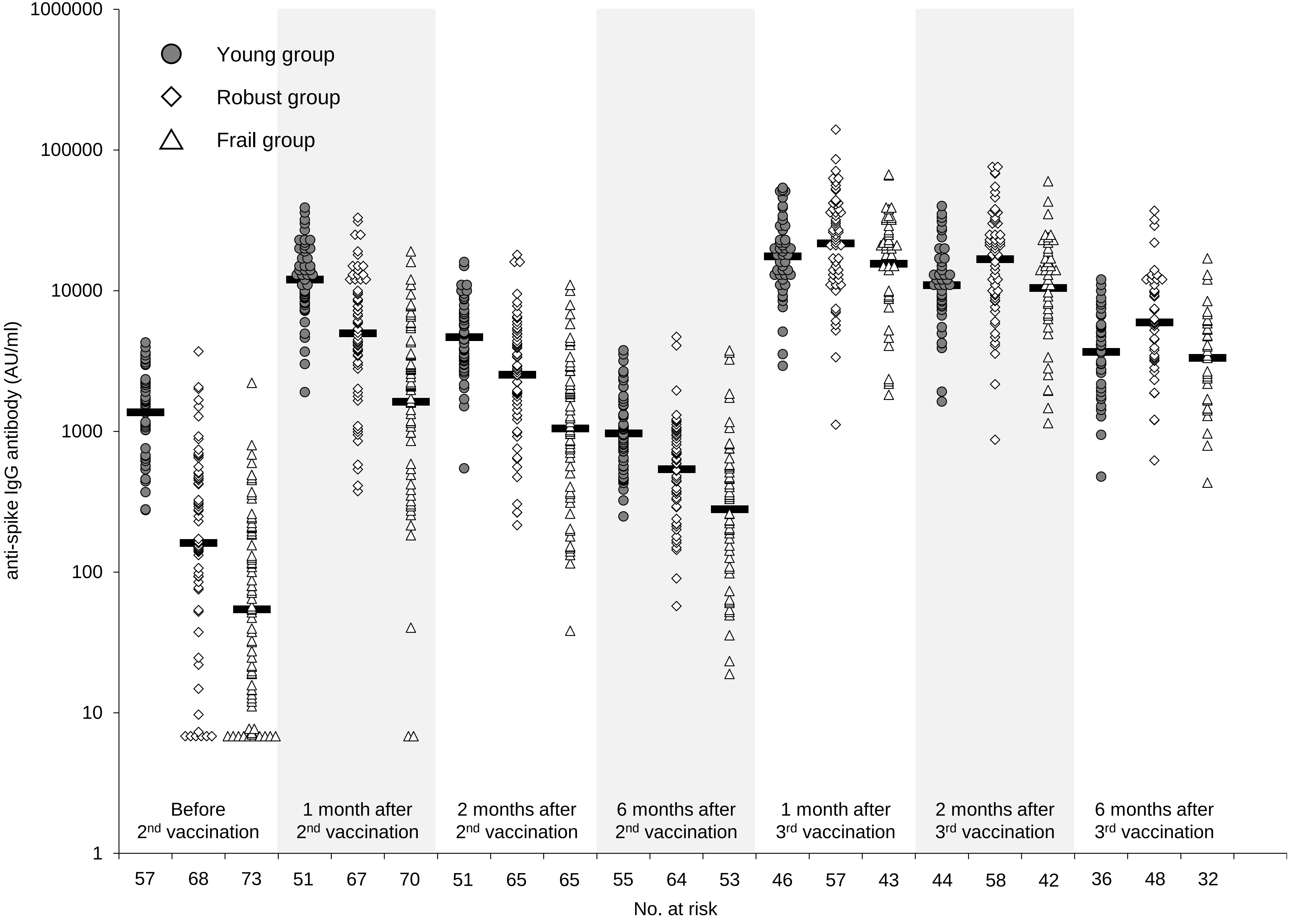

Figure S2. The correlations between cell-mediated immune responses and anti-spike IgG titers at one and two months after the third vaccination. (Related to Figure 1 and 2)

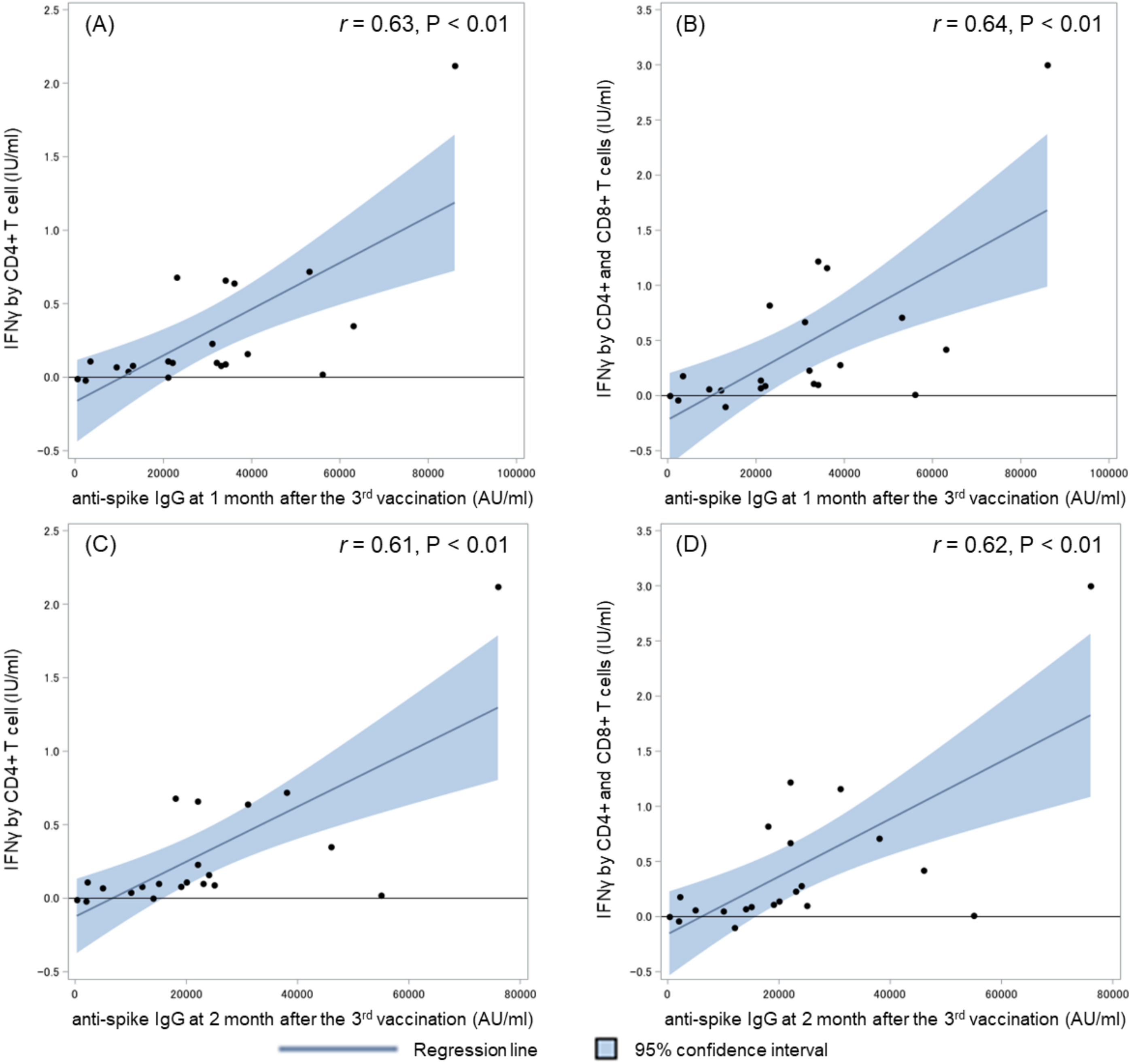

Figure S3. Flowchart of this study. (Related to Table 1 and 2)

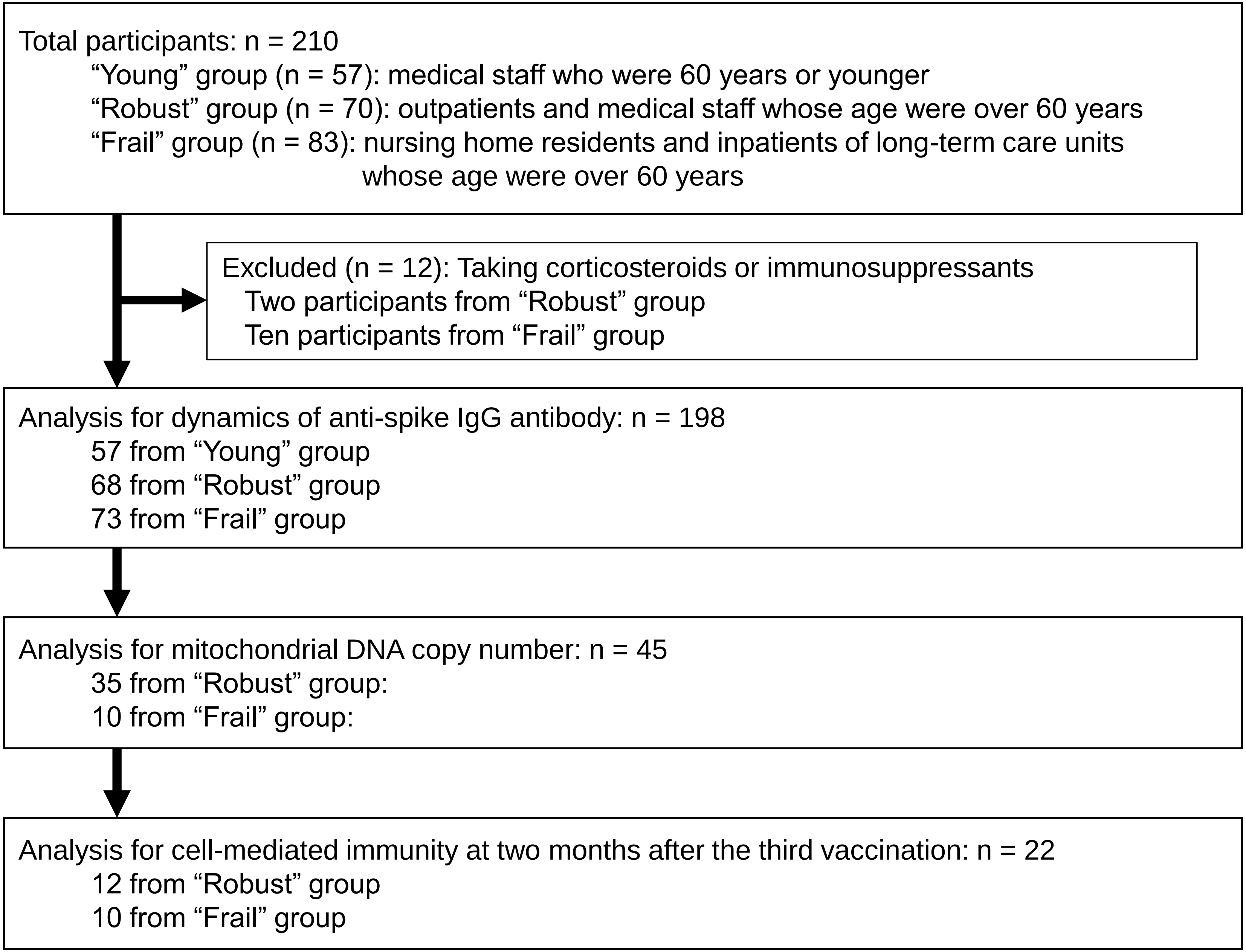

Supplemental Figure Legends

Figure S1. The dynamics of the anti-spike IgG titers across timepoints. (Related to Figure 1)

The dynamics of the anti-spike IgG titer of each participant at each timepoint (gray circles for the Young group, white squares for the Robust group, and white triangles for the Frail group) and geometric mean titer of anti-spike IgG (black bars) are shown.

Figure S2. The correlations between cell-mediated immune responses and anti-spike IgG titers at one and two months after the third vaccination. (Related to Figure 1 and 2)

Black circles represented each participant. The solid blue line shows the regression line, and the light blue area shows the 95% confidence interval. Figure S3A and S3B show the correlation between anti-spike IgG titers and interferon γ levels induced by CD4+ T cell (S3A) and CD4+ and CD8+ T cells (S3B) one month after the third vaccination. Figure S3C and S3D show the correlation between anti-spike IgG titers and interferon γ levels induced by CD4+ T cell (S3C) and CD4+ and CD8+ T cells (S3D) two months after the third vaccination. Interferon γ levels were measured two months after the third vaccination.

Figure S3. Flowchart of this study. (Related to Table 1 and 2)

Table S1. Baseline characteristics of participants in the anti-spike IgG dynamics analysis. (Related to Figure 1)

	Young medical staff (n=57)	Robust elderly† (n=68)	Frail elderly‡ (n=73)
Demographic			
Age – years	38 [29, 47]	78 [72, 84]	90 [86, 93]
Sex – no. (%)			
Female	53 (93.0)	40 (58.8)	56 (76.7)
Male	4 (7.0)	28 (41.2)	17 (23.3)
Smoking habits – no. (current / past / never)	0 / 5 / 52	3 / 14 / 51	0 / 3 / 70
Alcohol drinking habits – no. (daily / often / never)	5 / 32 / 20	14 / 16 / 38	2 / 2 / 69
Allergy – no. (%)	11 (19.3)	6 (8.8)	10 (13.7)
Comorbidities			
Number of comorbidities – no.	0 [0, 1]	3 [2, 4]	3 [1, 4]
Hypertension – no. (%)	4 (7.0)	45 (66.2)	56 (76.7)
Diabetes – no. (%)	2 (3.5)	29 (42.6)	19 (26.0)
Dyslipidemia – no. (%)	4 (7.0)	27 (39.7)	13 (17.8)
Hyperuricemia – no. (%)	1 (1.8)	11 (16.2)	7 (9.6)
Coronary heart disease – no. (%)	0 (0.0)	9 (13.2)	9 (12.3)
Arrhythmia – no. (%)	2 (3.5)	9 (13.2)	9 (12.3)
Stroke – no. (%)	0 (0.0)	5 (7.4)	26 (35.6)
Lung diseases – no. (%)	5 (8.8)	4 (5.9)	4 (5.5)
Thyroid disease – no. (%)	2 (3.5)	6 (8.9)	7 (9.6)
Osteoporosis – no. (%)	0 (0.0)	10 (14.7)	19 (26.0)
Autoimmune diseases – no. (%)	0 (0.0)	7 (10.3)	3 (4.1)
Cancer – no. (%)	0 (0.0)	8 (11.8)	15 (20.6)

Laboratory measurement			
Total bilirubin – mg/dl	0.6 [0.5, 0.8]	0.6 [0.5, 0.8]	0.5 [0.3, 0.6]
Aspartate aminotransferase – IU/ml	19 [15, 22]	24 [20, 28]	19 [16, 23]
Alanine aminotransferase – IU/ml	14 [11, 19]	18 [14, 26]	11 [9, 14]
γ-glutamyl transpeptidase – IU/ml	16 [14, 22]	24 [19, 35]	19 [14, 26]
Serum creatinine – mg/dl	0.60 [0.56, 0.67]	0.78 [0.64, 0.98]	0.75 [0.59, 0.94]
eGFR – ml/min/m^2	91.2 [79.1, 104.5]	62.0 [51.6, 71.7]	57.8 [44.3, 77.8]

† Robust elderly group consists of outpatients and medical staff who are older than 60 years and have independent ADL.

‡ Frail elderly group consists of nursing home residents and inpatients of long-term care units.

ADL, activities of daily living; eGFR, estimated glomerular filtration rate; IU, international unit.
